# Early-onset obesity dysregulates pulmonary adipocytokine/insulin signaling and induces asthma-like disease in mice

**DOI:** 10.1038/srep24168

**Published:** 2016-04-18

**Authors:** Katharina Dinger, Philipp Kasper, Eva Hucklenbruch-Rother, Christina Vohlen, Eva Jobst, Ruth Janoschek, Inga Bae-Gartz, Silke van Koningsbruggen-Rietschel, Christian Plank, Jörg Dötsch, Miguel Angel Alejandre Alcázar

**Affiliations:** 1 Experimental Pulmonology, University Hospital for Pediatrics and Adolescent Medicine, Faculty of Medicine, University of Cologne, Cologne, Germany; 2 Metabolism and Perinatal Programming, University Hospital for Pediatrics and Adolescent Medicine, Faculty of Medicine, University of Cologne, Cologne, Germany; 3 Department of Pediatrics and Adolescent Medicine, University Hospital Erlangen, Erlangen, Germany; 4 Pediatric Pulmonology, University Hospital for Pediatrics and Adolescent Medicine, Faculty of Medicine, University of Cologne, Cologne, Germany; 5 University Hospital for Pediatrics and Adolescent Medicine, Faculty of Medicine, University of Cologne, Cologne, Germany

## Abstract

Childhood obesity is a risk factor for asthma, but the molecular mechanisms linking both remain elusive. Since obesity leads to chronic low-grade inflammation and affects metabolic signaling we hypothesized that postnatal hyperalimentation (pHA) induced by maternal high-fat-diet during lactation leads to early-onset obesity and dysregulates pulmonary adipocytokine/insulin signaling, resulting in metabolic programming of asthma-like disease in adult mice. Offspring with pHA showed at postnatal day 21 (P21): (1) early-onset obesity, greater fat-mass, increased expression of IL-1β, IL-23, and Tnf-α, greater serum leptin and reduced glucose tolerance than Control (Ctrl); (2) less STAT3/AMPKα-activation, greater SOCS3 expression and reduced AKT/GSK3β-activation in the lung, indicative of leptin resistance and insulin signaling, respectively; (3) increased lung mRNA of *IL-6, IL-13, IL-17A* and *Tnf-α*. At P70 body weight, fat-mass, and cytokine mRNA expression were similar in the pHA and Ctrl, but serum leptin and IL-6 were greater, and insulin signaling and glucose tolerance impaired. Peribronchial elastic fiber content, bronchial smooth muscle layer, and deposition of connective tissue were not different after pHA. Despite unaltered bronchial structure mice after pHA exhibited significantly increased airway reactivity. Our study does not only demonstrate that early-onset obesity transiently activates pulmonary adipocytokine/insulin signaling and induces airway hyperreactivity in mice, but also provides new insights into metabolic programming of childhood obesity-related asthma.

Childhood obesity has become an emerging epidemic in the last decade. Today about 32% of US children are either already overweight or at risk to become overweight^1^,^2^. Studies have shown that maternal obesity and early postnatal overfeeding promote early-onset overweight in children and represent thereby a major risk factor for obesity and obesity-associated diseases beyond infancy^3^,^4^. Moreover, children with overweight or obesity are more likely to develop asthma compared to children with normal weight^5^. On the other hand, recent studies have identified several major risk factors for asthma^6^, including childhood obesity^7^. Clinical studies hypothesized that this increase of childhood obesity and asthma is not purely coincidental, but may, in fact, be causal^5^. According to the World Health Organization, more than 230 million people currently suffer from asthma and the incidence is rising, but no effective preventive strategies are developed to date.

Metabolic stress, either due to malnutrition or overnutrition, applied during critical windows of development, permanently alters physiology and metabolism of an organism by a phenomenon called metabolic programming^3^,^8^,^9^,^10^,^11^. For example, maternal obesity and early postnatal hyperalimentation (pHA) predispose the offspring for lifelong obesity with increase of adipose tissue, accompanied by impaired glucose tolerance, and features of metabolic syndrome^12^,^13^.

Adipose tissue is an active endocrine organ, producing a variety of inflammatory cytokines. These adipose tissue-originated cytokines, so called adipocytokines, initiate and aggravate inflammatory processes, which in turn can cause or exacerbate obesity-associated pathologies^14^,^15^,^16^,^17^. Notable among those adipocytokines are leptin, interleukin-6 (IL-6), IL-1β, and IL-23. They have been shown to induce chronic low-grade inflammation in various organs^17^,^18^, and are linked with the pathogenesis of lung disease^19^. For example, leptin has been linked to body weight-gain associated asthma by modulating lung injury and repair^20^,^21^. Furthermore, IL-6, IL-1β, and IL-23 do not only exert a pro-inflammatory function, but also influence development of the lung and lung diseases, ultimately favouring asthmatic phenotype in adulthood^22^,^23^,^24^,^25^.

Hyperinsulinemia in conjunction with disrupted insulin signaling, is a key feature of obesity^26^. Insulin is a pleiotropic hormone regulating glucose and lipid metabolism as well as growth and differentiation of cells^27^. It is recognized as a potential key modulator of lung development and lung disease by promoting growth and proliferation of cells within the lung, such as airway smooth muscle cells and also by increasing airway contractility and production of contractile proteins^28^,^29^,^30^,^31^,^32^.

Based on this evidence, we hypothesized that transient early postnatal hyperalimentation during a critical window of lung development induces early-onset obesity, dysregulates pulmonary adipocytokine and insulin signaling, and leads to airway hyperreactivity and asthma-like disease in “non-overweight” adult mice. In order to induce early postnatal overweight, early-onset obesity and increased white adipose tissue in the offspring at postnatal day 21 (P21) the dams were fed high-fat diet (HFD) during lactation from P1 to P21 (early postnatal hyperalimentation, pHA), whereas the Ctrl received standard diet. After weaning at P21 we fed the pHA-group and the Ctrl with standard diet, which led to similar body weight of both groups at P70. Despite similar body weight of both groups in adulthood, mice after pHA exhibited a marked airway hyperreactivity in comparison to the Ctrl, which could mechanistically be linked to a transient dysregulation of adipocytokine/insulin signaling during late lung development as found in the pHA-group. This study design gives the opportunity to analyze the effect of transient pHA-induced obesity (P21) on metabolic programming of lung function in adulthood (P70) by evaluating adipose tissue-originated proinflammatory cytokines, insulin signaling and extracellular matrix (ECM) proteins in the lung.

## Results

### Maternal HFD during lactation induces early postnatal hyperalimentation, early-onset overweight and increased white adipose tissue (WAT) in the murine offspring

**Figure 1:**
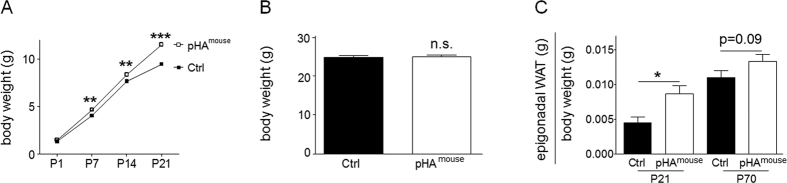
Maternal high-fat diet (HFD) feeding during lactation induces early postnatal hyperalimentation (pHA), early-onset overweight and increased white adipose tissue (WAT) in the murine offspring. (**A**) Body weight gain (gram) from postnatal day 1 (P1) to P21. Early postnatal hyperalimentation (open square; n = 36 from 9 litters; pHA^mouse^ group), Ctrl (solid square; n = 30 from 6 litters; Ctrl). (**B**) Body weight (gram) at P70 (Ctrl: n = 21 from 7 litters; pHA^mouse^: n = 18 from 6 litters). (**C**) WAT weight (gram) relative to body weight (gram) at P21 (Ctrl: n = 10 from 5 litters; pHA^mouse^: n = 11 from 6 litters) and at P70 (Ctrl: n = 11 from 6 litters; pHA^mouse^: n = 17 from 7 litters). pHA^mouse^ group: white bar, Ctrl: black bar. Mean ± SEM; two way ANOVA test and Bonferroni posttest (**A**), Mann Whitney test. (**B,C**); *p < 0.05, **p < 0.01, ***p < 0.001; n.s. = not significant.

To analyze the effect of maternal HFD during lactation on the body weight of the offspring, body weight was determined at P1, P7, P14 and P21 (Fig. 1A). The body weight of the pHA^mouse^ group was greater than in the Ctrl at P21 (p < 0.001) (Fig. 1A), whereas it was unchanged at P70 (Fig. 1B). Furthermore, WAT relative to body weight was greater in the pHA^mouse^ group than in the Ctrl at P21 (p < 0.05) and tended to be higher at P70 (Fig. 1C). In summary, early pHA leads to transient early-onset overweight and obesity at P21; whereas at P70 body weight and WAT is similar to Ctrl.

To validate our data and exclude a species- and maternal-diet dependent effect we used a second model in rat, in which we induced early postnatal overweight in the offspring by litters size reduction at birth. The rat model of pHA and the relevant results are described in detail in the supplementary online data.

### Adipose tissue-originated cytokines, adipocytokines, are greater in mice with early-onset overweight

**Figure 2:**
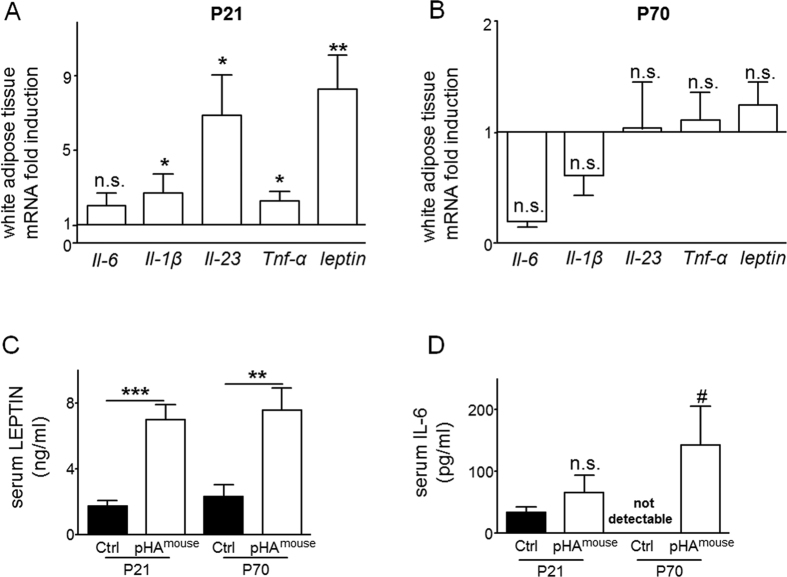
Early postnatal hyperalimentation (pHA) and early-onset obesity lead to transient increased mRNA expression of adipocytokines and persistent greater circulating concentrations of serum leptin and IL-6 in murine offspring. (**A,B**) Total white adipose tissue (WAT) mRNA expression of genes encoding *Il-1β*, *Il-6*, *Il-23, Tnf-α, and leptin* was assessed by quantitative real-time PCR at postnatal day 21 P21 (**A**) (Ctrl: n = 9 from 5 litters; pHA^mouse^: n = 11 from 6 litters) and P70 (**B**) (Ctrl: n = 9 from 6 litters; pHA^mouse^: n = 9 from 6 litters). The Ctrl was normalized to 1; early postnatal hyperalimentation (white bar; pHA^mouse^ group). (**C**) Serum level of leptin (ng/ml) at P21 (Ctrl: n = 8 from 5 litters; pHA^mouse^: n = 8 from 6 litters) and at P70 (Ctrl: n = 7 from 6 litters; pHA^mouse^: n = 7 from 4 litters). (**D**) Serum level of IL-6 (pg/ml) at P21 (Ctrl: n = 7 from 5 litters; pHA^mouse^: n = 5 from 5 litters) and at P70 (Ctrl: n = 4 from 3 litters; pHA^mouse^: n = 5 from 2 litters). pHA^mouse^ group: white bar, Ctrl: black bar. Mean ± SEM; Mann Whitney test (*), two way ANOVA test and Bonferroni posttest (#); *^,#^p < 0.05, **p < 0.01, ***p < 0.001; n.s. = not significant.

To investigate the effect of the increase of WAT on the level of adipose tissue-originated cytokines, we assessed expression of genes encoding *interleukin-1β* (*Il-1β)*, *Il-6*, *Il-23*, tumor necrosis factor-α (*Tnf-α*), and *leptin* in WAT at P21 and P70. The mRNA expression of *Il-1β* (p < 0.05), *Il-23* (p < 0.05), *Tnf-α* (p < 0.05), and *leptin* (p < 0.05) was increased at P21 (Fig. 2A), whereas at P70 no significant changes were found in the pHA-group, when compared to Ctrl (Fig. 2B).

Moreover, we also measured serum concentrations of leptin and IL-6 using ELISA and found a significant increase of serum leptin (3-fold induction; p < 0.001; Fig. 2C) and slightly higher IL-6 concentrations (Fig. 2D) in the pHA^mouse^ group in comparison to the Ctrl-group; at P70 both concentrations of leptin (p < 0.01; Fig. 2C) and of IL-6 (p < 0.05; Fig. 2D) were significantly elevated after pHA when compared to Ctrl.

In summary, gene expression of cytokines in the WAT is transiently increased after pHA and the elevation of circulating leptin and IL-6 persists into adulthood.

### Greater expression of pro-asthmatic cytokines and dysregulation of STAT3-AMPKα-SOCS3 signaling in lungs of mice with early-onset overweight

**Figure 3:**
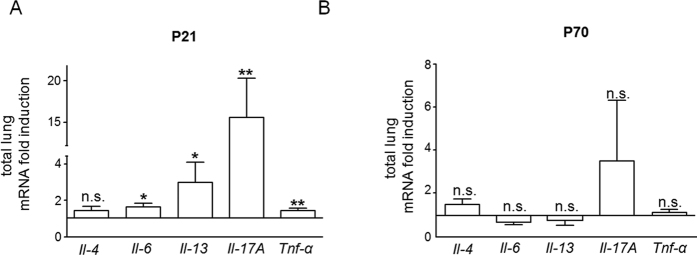
Greater expression of pro-asthmatic cytokines (IL-4, IL-6, IL-13, IL-17A, and TNFα) in lungs of mice with early postnatal hyperalimentation (pHA) and early-onset obesity. (**A,B**) Total lung mRNA expression of genes encoding *Il-4*, *Il-6*, *Il-13*, *Il-17A* and *Tnf-α* was assessed by quantitative real-time PCR at (A) P21 (Ctrl: n = 10 from 5 litters; pHA^mouse^: n = 10 from 6 litters) and at (**B**) P70 (Ctrl: n = 9–10 from 6 litters; pHA^mouse^: n = 10 from 6 litters). The Ctrl was normalized to 1; early postnatal hyperalimentation (white bar; pHA^mouse^group). Mean ± SEM; Mann Whitney test; *p < 0.05, **p < 0.01; n.s. = not significant.

We measured pulmonary mRNA of *Il-1β, Il-4, Il-6, Il-13, Il-17A, Il-23 and TNF-α* at postnatal day 21 (P21) and P70, and found a significant greater expression of *Il-6* (p < 0.05), *Il-13* (p < 0.05), *Il-17A* (p < 0.01) and *Tnf-α* (p < 0.01), but not of *Il-1β* and *Il-23* (data not shown) in pHA^mouse^ at P21 than in Ctrl (Fig. 3A); whereas at P70, no significant differences were detectable (Fig. 3B).

**Figure 4:**
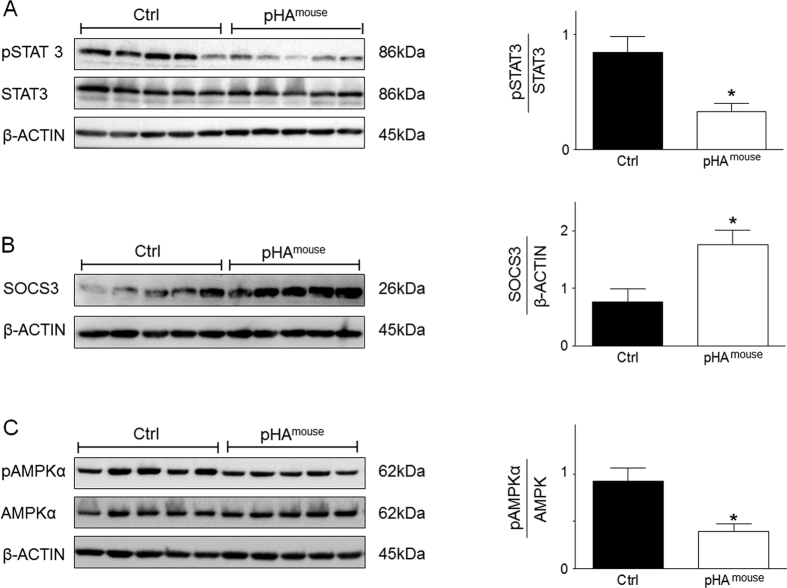
Early dysregulation of STAT3-AMPKα-SOCS3 signaling in lungs of mice with early postnatal hyperalimentation (pHA) and early-onset obesity at postnatal day 21 (P21). (**A**) Immunoblots showing lung protein expression of total STAT3, and phosphorylation of STAT3 (pSTAT3) at P21; Ctrl: n = 5 from 4 litters; pHA^mouse^: n = 5 from 4 litters. (**B**) Assessment of suppressor of cytokine signaling 3 (SOCS3), a leptin/IL-6 target, by immunoblot at P21; Ctrl: n = 5 from 4 litters; pHA^mouse^: n = 5 from 4 litters. (**C**) Representative immunoblot for total AMPKα and phosphorylated AMPKα (p AMPKα) in lungs at P21; Ctrl: n = 6 from 4 litters; pHA^mouse^: n = 8 from 4 litters. β-ACTIN served as loading Control (Ctrl). The densitometric analyses are presented next to the respective immunoblot. pHA^mouse^group: white bar; Ctrl: black bar. Mean ± SEM; Mann Whitney test; *p < 0.05.

Next, we analyzed the intrinsic pulmonary leptin- and IL-6 signaling using phosphorylation of the signal transducer and acticvator of transcription 3 (pSTAT3) as an indicator. Phosphorylated STAT3 in lungs of the pHA^mouse^ group was significantly reduced when compared to Ctrl (p < 0.05) (Fig. 4A). Suppressor of cytokine signaling 3 (SOCS3) as a target of both leptin and IL-6 signaling exerts a regulatory feedback function by inhibiting activation of STAT3 signaling. Assessment of protein abundance showed a significant greater increase of SOCS3 in lungs of the pHA^mouse^ group than in Ctrl at P21 (p < 0.05) (Fig. 4B). Phosphorylation of AMP-activated protein kinase α (AMPKα) is downstream of STAT3 signaling and intial studies suggest that SOCS3 could also inhibit STAT3-mediated AMPKα activation^33^. We found a significant reduction of phosphorylated AMPKα in lungs at P21 after pHA when compared to Ctrl (p < 0.05; Fig. 4C). In summary, early-onset overweight increases pulmonary expression of pro-ashmatic cytokines and markers of Th17 cells, and increases SOCS3 expression coupled with inhibition of STAT3/AMPKα activation at P21.

### Early pHA induces glucose intolerance and dysregulates intrinsic pulmonary insulin signaling in murine lung

**Figure 5:**
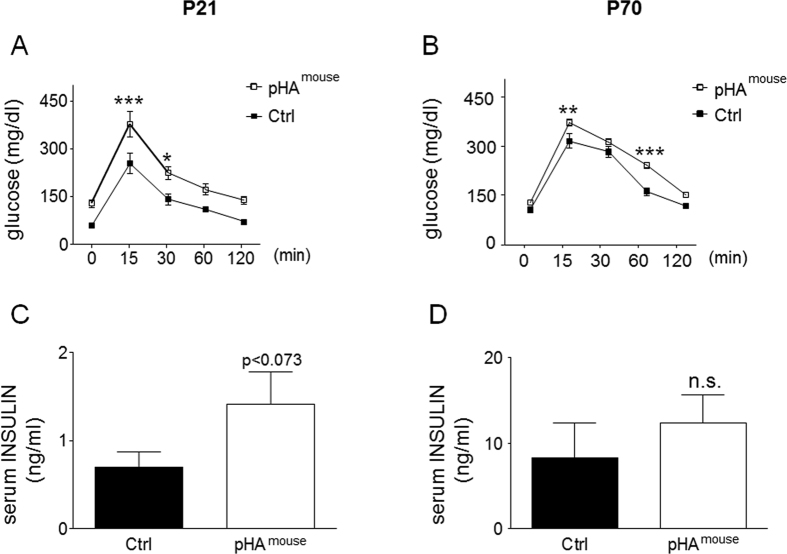
Early-onset overweight with early-onset obesity induces glucose intolerance and mild hyperinsulinemia. (**A,B**) Intraperitoneal glucose tolerance test (i.p. GTT) at postnatal day 21 (**A**) and 70 (**B**) (P21 and P70); blood glucose levels (mg/dl) 0, 15, 30, 60, and 120 min after i.p. injection of glucose. P21: Ctrl, n = 12 from 5 litters; pHA^mouse^: n = 6 from 6 litters: P70: Ctrl: n = 7 from 3 litters; pHA^mouse^: n = 16 from 5 litters. two way ANOVA test and Bonferroni posttest. Early postnatal hyperalimentation (open square; pHA^mouse^group), Ctrl (solid square; Ctrl). (**C,D**) Serum levels of insulin (ng/ml) at P21 (**C**): Ctrl: n = 7 from 5 litters; pHA^mouse^: n = 7 from 6 litters, and P70 (**D**): Ctrl: n = 5 from 4 litters; pHA^mouse^, n = 6 from 3 litters. Mann Whitney test; *p < 0.05, **p < 0.01, ***p < 0.001; n.s. = not significant.

To clarify whether the increase of adipocytokines is related to impaired glucose metabolism we tested glucose tolerance by assessing intraperitoneal glucose tolerance test (i.p. GTT). At P21, pHA^mouse^ showed significantly greater serum glucose levels compared to Ctrl at 15 min (p < 0.001) and 30 min (p < 0.05) following i.p. glucose injection (Fig. 5A). At P70, glucose tolerance was partially restored compared to P21, but serum glucose was still higher at 15 min (p < 0.01) and 60 min (p < 0.001) in the pHA^mouse^ group when compared to the Ctrl (Fig. 5B). Insulin in serum was greater in the pHA^mouse^ group than in the Ctrl at P21 (p = 0.073 by Mann Whitney test; Fig. 5C), but not at P70 (Fig. 5D).

**Figure 6:**
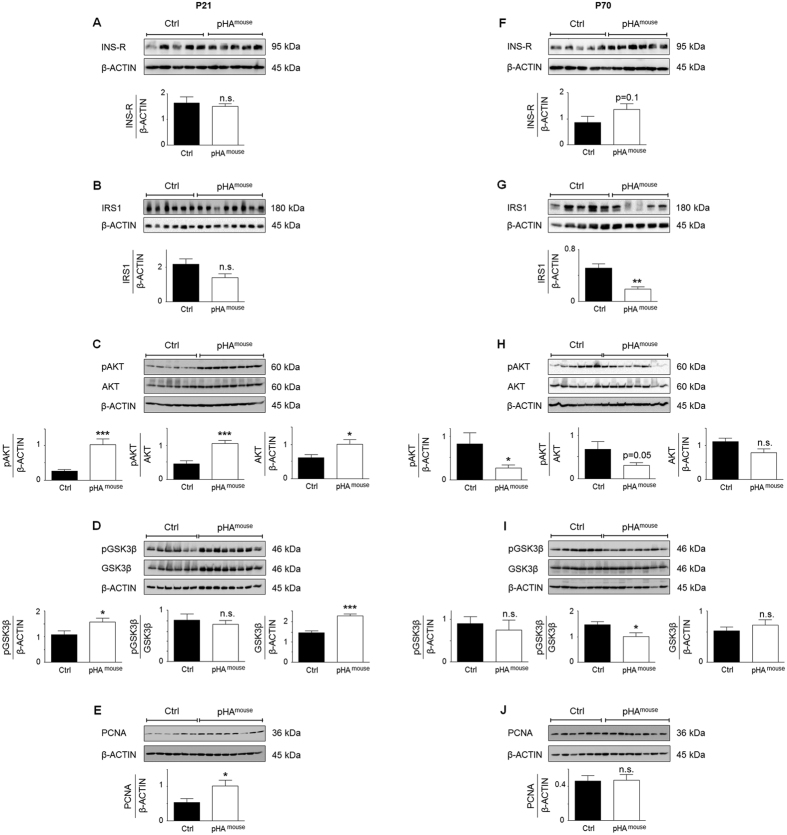
Early postnatal hyperalimentation (pHA) with early-onset obesity induces transient activation of intrinsic pulmonary insulin signaling at postnatal day 21 (P21), and inhibition at P70. (**A–E**) Immunoblots showing indicators of insulin signaling at P21. (**A**) Protein expression of insulin receptor (INS-R) in lungs at P21; Ctrl: n = 5 from 4 litters; pHA^mouse^: n = 5 from 4 litters. (**B**) Protein abundance of insulin receptor substrate 1 (IRS1) in lungs at P21; Ctrl: n = 6 from 4 litters; pHA^mouse^: n = 8 from 4 litters. (**C**) Assessment of phosphorylated AKT (pAKT) and total AKT in the lungs at P21; Ctrl: n = 6 from 4 litters; pHA^mouse^: n = 8 from 4 litters. (**D**) Immunoblots showing phosphorylated and total GSK-3β in lungs at P21; Ctrl: n = 6 from 4 litters; pHA^mouse^: n = 8 from 4 litters. (**E**) Protein abundance of proliferating cell nuclear antigen (PCNA) as an index of proliferation at P21; Ctrl: n = 6 from 4 litters; pHA^mouse^: n = 8 from 4 litters. (**F–J**) Assessement of indicators of insulin signaling at P70 using immunoblots. (**F**) Protein expression of INS-R in the lungs at P70; Ctrl: n = 5 from 4 litters; pHA^mouse^: n = 5 from 4 litters. (**G**) Protein abundance of IRS1 in lungs at P70; Ctrl: n = 5 from 4 litters; pHA^mouse^: n = 5 from 4 litters. (**H**) Immunoblots for pAKT and total AKT in lungs at P70; Ctrl: n = 6 from 4 litters; pHA^mouse^: n = 8 from 4 litters. (**I**) Phosphorylated and total GSK-3β in lungs at P21; Ctrl: n = 6 from 4 litters; pHA^mouse^: n = 8 from 4 litters. (**J**) Immunoblots showing PCNA at P70; Ctrl: n = 6 from 4 litters; pHA^mouse^: n = 8 from 4 litters. Densitometric analyses were performed and are shown below the corresponding immunoblot; β-ACTIN served as loading Control (Ctrl). Mann Whitney or unpaired t-test. pHA^mouse^group: white bar; Ctrl: black bar. Mean ± SEM; *p < 0.05, **p < 0.01, ***p < 0.001; n.s. = not significant.

Measurement of total insulin receptor (INS-R) and insulin receptor substrate 1 (IRS1) using immunoblot did not show any significant differences between pHA^mouse^ and Ctrl at P21 (Fig. 6A,B). We next analyzed the intrinsic pulmonary insulin pathway by assessing AKT and glycogen synthase kinase 3β (GSK-3β) signaling as indicators of intracellular insulin signaling. GSK-3β is phosphorylated at Ser-9 and thereby inactivated by phosphorylated Akt. We found that total protein abundance of AKT (p < 0.05) and GSK3β (p < 0.001) and also the phosphoryalation of both (p < 0.001 and p < 0.05, respectively) were markedly increased after pHA at P21 when compared to the Ctrl (Fig. 6C,D). Since insulin is known as a proliferative signaling pathway we assessed proliferating cell nuclear antigen (PCNA) as an indicator of proliferation and found a significant increase in lungs of the pHA^mouse^-group at P21 (Fig. 6E). At P70, however, we detected a slightly higher protein abundance of INS-R (p = 0.1; Fig. 6F), reduced expression of IRS1 (p < 0.01) (Fig. 6G), lower phosphorylated AKT (p < 0.05) (Fig. 6H), and reduced phosphorylated GSK-3β related to total GSK-3β. Assessment of PCNA did not show any differences between pHA^mouse^-group and Ctrl at P70 (Fig. 6J).

In summary, transient early-onset obesity results in glucose intolerance at P21 and P70. Markers of insulin signaling indicate an activation of intrinsic pulmonary insulin signaling at an early phase (P21) and features of insulin resistance at a late phase (P70).

### Early pHA with early-onset overweight regulates murine pulmonary soluble elastin and leads to greater collagen IαI protein abundance in lungs of murine offspring

**Figure 7:**
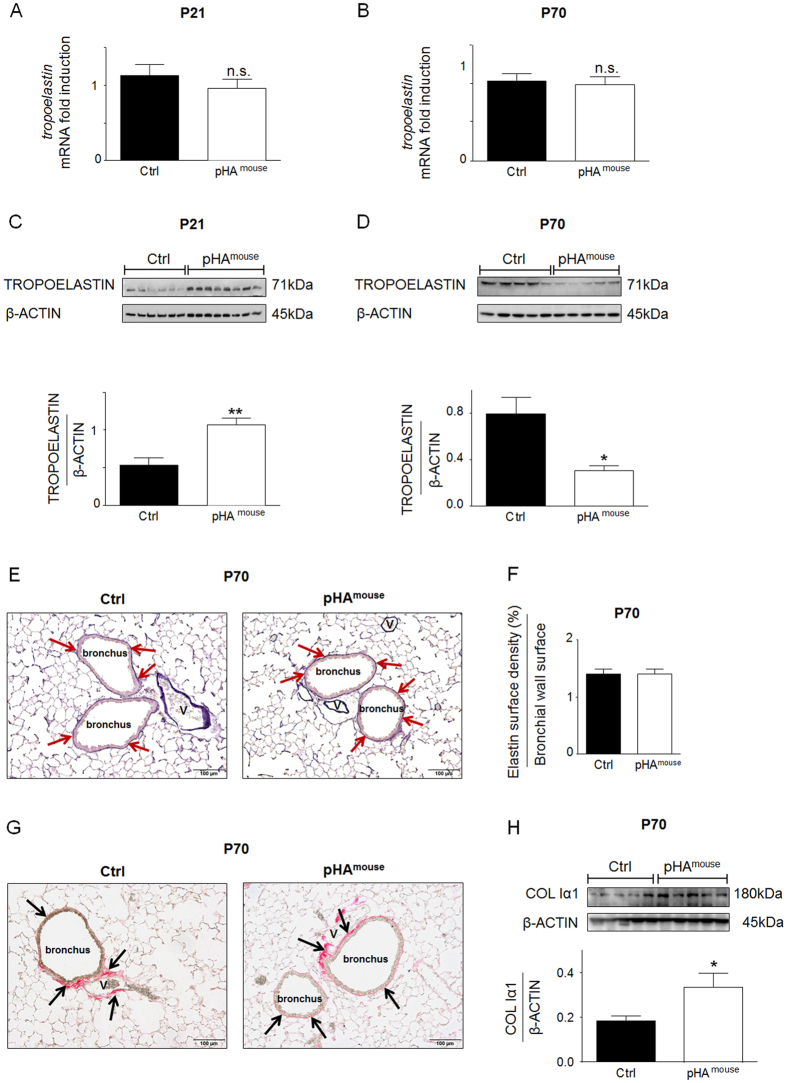
Early postnatal hyperalimentation (pHA) with early-onset obesity temporo dynamically regulates murine pulmonary elastin synthesis and induces greater collagen Iα1 protein abundance. (**A,B**) Assessment of *tropoelastin* mRNA in total lung homogenate by quantitative qRT-PCR at postnatal day 21 (P21) (**A**) (Ctrl: n = 10 from 5 litters; pHA^mouse^: n = 10 from 6 litters) and at P70 (**B**) (Ctrl: n = 9–10 from 6 litters; pHA^mouse^: n = 10 from 6 litters). (**C,D**) Lung protein abundance of TROPOELASTIN at P21 (**C**) (Ctrl: n = 6 from 4 litters; pHA^mouse^: n = 8 from 4 litters) and at P70 (**D**) (Ctrl: n = 5 from 4 litters; pHA^mouse^: n = 5 from 4 litters). β-ACTIN served as loading Control (Ctrl). Densitometric analyses below the corresponding immunoblot. (**E**) Representative images illustrating elastic fibers using Hart’s staining in paraffin-embedded and paraformaldehyde-fixed lungs of the Ctrl-group (left panel) and of the pHA^mouse^-group (right panel) at P70. Red arrows are depicting positive staining of elastic fibers of the conducting airways (100–200 μm diameter). (**F**) Summary data of the quantification of positive elastic fiber staining surrounding the bronchi (100–200 μm) at P70. Elastin surface density was related to bronchial wall surface; pHA^mouse^ group: n = 6 from 4 litterss, Ctrl: n = 6 from 5 litterss. (**G**) Representative images illustrating sirius-red staining used to visualize connective tissue of the peribronchial area (black arrows); pHA^mouse^ group: n = 6 from 4 litterss, Ctrl: n = 6 from 5 litterss. (**H**) Immunoblot showing protein abundance of collagen Iα1 (COL Iα1) at P70. β-ACTIN served as loading Control (Ctrl); Ctrl: n = 5 from 4 litters; pHA^mouse^: n = 5 from 4 litters. Early postnatal hyperalimentation (pHA^mouse^ group) compared to the Ctrl (Ctrl). pHA^mouse^ group: white bar; Ctrl: black bar. Mean ± SEM; Mann Whitney test. *p < 0.05, **p < 0.01; n.s. = not significant.

To analyze whether transient early-onset overweight along with a transient activation of adipocytokine and insulin signaling is related to long-term changes of the expression of key ECM molecules, we assessed expression of tropoelastin at P21 and P70. Even though gene expression was not significantly changed at P21 (Fig. 7A) or P70 (Fig. 7B), protein abundance of tropoelastin in total lung homogenate was markedly increased at P21 (p < 0.01), but decreased at P70 (p < 0.05) (Fig. 7C,D). The process of elastic fiber formation and assembly occurs during development and the fibers are present throughout life. Some studies indicate that there is a reduction of elastic fibers in the ageing lung^34^,^35^. Since elastin is an important component of elastic fibers, main regulator of elasticity of the lung, we performed Hart’s stain using tartrazine as counterstain. Neither representative images depicting peribronchial elastic fibers as indicated by red arrows in lungs at P70 (Fig. 7E) nor the corresponding quantification (Fig. 7F) showed significant differences between pHA^mouse^ and Ctrl-group.

In addition, to determine whether transient obesity induces increased protein abundance of pro-fibrotic markers we measured collagen Iα1 by immunoblot and found a marked increase after pHA at P70 (p < 0.05) (Fig. 7H). Representative sirius-red staining of conducting airways, (black arrows) however, does not indicate marked peribronchial fibrosis in lungs after pHA at P70 (Fig. 7G).

To summarize, early-onset obesity affects lung matrix remodeling by regulating tropoelastin expression and abundance as well as increasing collagen Iα1 production in lungs after pHA, potentially increasing thereby the susceptibility to pro-fibrotic changes in the pHA-group.

### Early pHA with early-onset overweight leads to airway hyperreactivity in the murine model at P70, but does not alter bronchial structure or alveolar size

**Figure 8:**
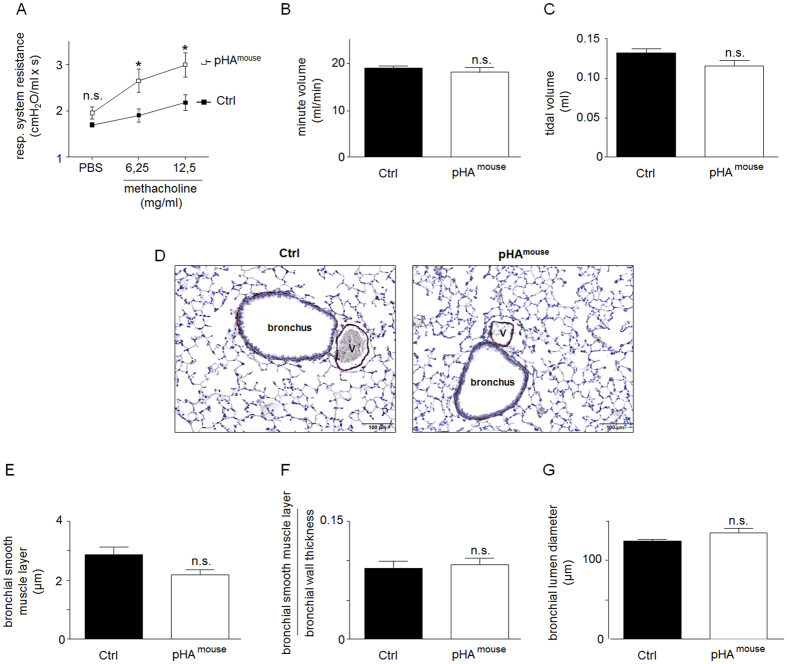
Early postnatal hyperalimentation pHA with early-onset obesity leads to greater airway resistance in murine model at postnatal day (P70) despite unaltered bronchial structure. (**A**) Airway resistance (Res) after methacholine stimulation measured by direct plethysmography. Exposure of the mice at postnatal day 70 (P70) to PBS, followed by methacholine: 6.25 mg/ml and 12.5 mg/ml. Res was significantly increased after methacholine stimulation at P70 in the group with early postnatal hyperalimentation (pHA^mouse^group, n = 14 from 4 litters; open square) compared to the Ctrl (Ctrl, n = 9 from 6 litters; solid square); two-way ANOVA and Bonferroni posttest. (**B**) Minute volume and (**C**) tidal volume were measured during direct plethysmography; Mann Whitney test. pHA^mouse^ group: white bar; Ctrl: black bar; Mean ± SEM; Significance for each bar is indicated by p values. (**D**) Representative images of bronchi stained for α-smooth muscle actin (α-SMA) as an indicator of smooth muscle cells. Summary data for (**E**) measurement of the thickness of the bronchial smooth muscle layer (bSML) in the Ctrl-group and in the pHA^mouse^-group at P70; (**F**) ratio of thickness of the bSML and bronchial wall thickness; (**G**) diameter of the lumen of the bronchi. Only bronchi with 100–200 μm diameter were used for assessment of bSML and bronchial wall thickness. pHA^mouse^group (n = 6 from 4 litterss): white bar; Ctrl (n = 6 from 5 litterss): black bar. Mean ± SEM; Mann Whitney test. *p < 0.05, **p < 0.01; n.s. = not significant.

To determine whether a dysregulation of insulin and adipocytokine signaling as a result of early pHA affects long-term lung function we assessed airway resistance (Res) in adulthood, performing direct plethysmography at P70. The pHA^mouse^ group exhibited a significant greater airway Res and airway hyperresponsiveness after methacholine stimulation when compared to the Ctrl-group (Fig. 8A). Minute volume (Fig. 8B) and tidal volume (Fig. 8C) were similar in the pHA^mouse^ group and Ctrl. Moreover, assessment of respiratory dynamic compliance (Cdyn) to determine elasticity of the lung did show a slight, but not significant increase (supplemental Fig. 1C).

We next investigated whether this airway hyperreactivity after pHA is related to long-term changes of bronchial structure and lung growth. Assement of the thickness of the bronchial smooth muscle layer (bSML), and calculation of the ratio of bSML and bronchial wall thickness was performed to evaluate bronchial structure, whereas mean linear intercept (MLI) was used as a parameter of alveolar size. We did not find differences in thickness of bSML, bSML related to bronchial wall thickness, and mean lumen diameter of the bronchi analyzed between the two groups, pHA^mouse^ (n =6 from 2 litterss) and Ctrl (n =6 from 2 litterss) (Fig. 8D–G). Moreover, MLI of lungs after pHA at P70 was similar to the Ctrl-group (supplemental Fig. 1A,B).

## Discussion

Here, we established a novel model of transient early-onset obesity induced by maternal HFD during lactation to investigate the role of nutrition in the early postnatal period on long-term lung function. In our study we tested the hypothesis that overweight in an early postnatal window leads to metabolic programming of asthma-like disease in adulthood. The findings demonstrate that a transient early postnatal overweight induces early-onset (P21) obesity, accompanied by activation of pulmonary adipocytokine/insulin signaling and increased pulmonary expression of pro-asthmatic IL-6, IL-13, IL-17A and Tnf-α. Those early-onset changes are ultimately related to long-term metabolic programming of airway hyperreactivity and ECM in the lung.

### Transient early-onset obesity induced by pHA is linked to long-term airway hyperreactivity

We analyzed two time points in our murine model of HFD-induced pHA: P21 resembling childhood, and P70 resembling adulthood. Early pHA results in an accelerated weight gain prior weaning and transient early-onset overweight at P21. Even though both groups have been fed with the same standard diet after weaning, the pHA^mouse^ group maintained a slight, but not significant increased epigonadal WAT. In contrast, the body weight was similar in both groups at P70, indicating that a transient overweight during a critical developmental window induce a persistent change of body composition favouring WAT accumulation. Clinical reports support these results by showing that children of obese mothers and with accelerated postnatal weight gain are more likely to develop increased body weight gain, obesity, and higher risk for asthma^4^,^13^. Epidemiological studies have shown that asthma outcomes ameliorate after dietary intervention and loss of body weight^36^. Intriguingly, our model revealed an increased airway resistance in adult rodents despite a dietary intervention and normalization of body weight, raising the question for “metabolic programming” of long-term lung function.

High-fat diet does also influence maternal metabolism and induce hormonal changes. Those changes could affect the composition of breast milk and contribute thereby to changes of the microbiome of the offspring and ultimately result in changes seen in our study^3^,^37^. Those aspects let us query whether the changes we see in our animal model are the result of altered breast milk or rather of increased caloric intake. To exclude a species- or maternal diet-dependent effect, we verified our results in a rat model of pHA by litters size reduction (see supplemental data). Indeed, similar to the murine model of maternal HFD-induced early-onset obesity, rats following litters size reduction exhibited early-onset overweight at P21 (supplemental Fig. 3A), but no differences in body weight at P70 compared to the Ctrl (supplemental Fig. 3B). Moreover, SOCS3 protein expression in the lung (supplemental Fig. 5) and airway reactivity (supplemental Fig. 4) were increased as well. These results confirm the findings of our murine model of pHA and demonstrate the impact of early postnatal caloric intake and early-onset obesity on “metabolic programming” of airway hyperreactivity.

### Elevated adipocytokines after early pHA are related to features of pulmonary leptin resistance

Clinical studies associate obesity with a chronic, low-grade systemic inflammation related to increased levels of adipocytokines, which are suggested to be mechanistically important in obesity-associated diseases^15^,^17^. For example, increased levels of serum leptin are linked with body weight-gain-associated asthma^21^,^38^; moreover, hyperleptinemia in lean mice increases airway resistance independent of Th_2_ cells, suggesting a key role of leptin linking obesity and asthma. In addition, treatment of obese mice with anti-IL-6 antibody reduces airway hyperresponsiveness^38^. In our study, on one hand we show only a transient early-onset overweight after pHA at P21 coupled with elevated serum levels of the aforementioned adipocytokines, indicative of an early low-grade inflammatory state. On the other hand, despite normalization of body weight following early-onset overweight after pHA these mice exhibit airway hyperreactivtiy and asthma-like phenotype in adulthood. Moreover, pHA-mice did not exhibit differences in body weight in adulthood, but the serum adipocytokine levels, such as leptin and IL-6, remained increased at P70, indicating persistent metabolic changes favouring inflammation and potentially contributing to the asthma-like phenotype in those mice as well. Leptin/ IL-6 signaling *via* STAT3 signaling has been reported to be a key regulator of inflammatory processes, and modulator of matrix remodelling and cell proliferation, and therefore potentially crucial in the pathogenesis of asthma^20^,^39^. IL-6 in induced sputum of patients evolving asthma is increased and correlated with FEV1^40^. The intracellular signaling of leptin and IL-6 occurs predominantly through the activation of STAT3 and AMPKα, which in turn can induce expression of SOCS3. In fact, SOCS3 is known as a target of leptin and IL-6 and exerts inhibitory function as a negative feedback on STAT3^41^. Various recent experimental studies highlighted the link of STAT3/SOCS3 and a pro-asthmatic phenotype. For example, STAT3 Protein has been shown to regulate vascular smooth muscle cell phenotypic switch^42^. Moreover, gene silencing of SOCS3 by siRNA *via* intranasal delivery inhibits asthma phenotype in mice and underlines the crucial role of SOCS3 in the pathogenesis of asthma^43^. Those experimental findings were confirmed in humans by polymorphisms in STAT3 and SOCS3, associated with asthma^44^,^45^. In our study, despite the increased circulating levels of leptin and IL-6 in the pHA^mouse^ group, we determined a significant inhibition of STAT3/AMPKα phosphorylation. It is known that obesity induces a leptin-resistance^46^ either through 1) a direct effect of leptin or 2) inflammatory cytokines, such as IL-6 in a leptin-independent manner^14^,^41^. Interestingly, in our study we demonstrate an increased expression of SOCS3, indicating a postreceptor leptin resistance in lungs of the pHA^mouse^ group and a link to asthmatic phenotype.

### Transient pro-asthmatic pulmonary cytokine profile after pHA: indicative of Th17 activation by adipocytokines

Obesity has been shown to modulate immune response and to be intimately linked with asthma. Recent studies by Kim *et al.* identified an unique role of IL-17A produced by innate lymphoid cells of the lung in mediating airway hyperreactivity in adult mice with HFD-induced obesity^47^,^48^. They showed elevated levels of IL-17A in lungs of obese mice with airway reactivity; in contrast IL-17^−/−^ mice were protected against impaired lung function following HFD. Moreover, IL-1β, IL-6 and IL-23 are known to induce IL-17A production in Th17 cells and are thereby linked to airway hyperreactivity related to obesity^49^. Lungs of humans with asthma have a characteristic expression pattern of inflammatory cytokines, notable amongst those IL-4, IL-6, IL-13, IL-17A and TNF-α^50^,^51^,^52^, indicative of Th_2_ cells and Th17 cells. Indeed, in our model at P21 we determined a marked increase of IL-1β, IL-23, and TNF-α in WAT after pHA; moreover, lung gene expression of IL-6, IL-13, and IL-17A was greater in the pHA^mouse^ group when compared to Ctrl, indicating that the expression pattern of inflammatory cytokines is shifted towards a pro-asthmatic pattern, favouring in part Th17 cells. Interestingly at P70, the time point of increased airway resistance, expression of these cytokines in WAT and lung were normalized. The transient increase of lung IL-17A could be mediated through transient circulating levels of adipose-tissue-originated IL-1β and IL-23, suggesting a WAT-lung axis in “metabolic programming” of asthma in adulthood.

### Temporo-dynamic regulation of insulin signaling following transient early-onset obesity

A chronic low-grade inflammatory status in obesity is known to induce impaired insulin signaling, glucose intolerance, and insulin resistance^53^. In our study we determined at P21 a low-grade inflammatory state with increased circulating adipocytokines. Those findings were related to impaired glucose tolerance, mild hyperinsulinemia, and activated AKT/GSK3β signaling. The reduced glucose uptake could be related to an insufficient increase of insulin secretion and insulin signaling. In contrast, greater phosphorylation of AKT/GSK3β suggest active insulin signaling and indicate that there could be an insulin-independent impairment of glucose tolerance. For example, SOCS3 has been associated with increased plasma insulin concentrations and glucose intolerance^33^,^54^. Therefore the increase of SOCS3 in the lungs of animals following pHA compared to Ctrl as shown in our study could contribute to the mild hyperinsulinemia and impaired glucose uptake after pHA at P21. At P70, however, we observed persistent impaired glucose tolerance coupled with reduced insulin signaling as shown by a decrease of phosphorylation of AKT and GSK3β, suggesting an insulin resistance in pHA^mouse^ compared to Ctrl as a consequence of metabolic programming induced by early-onset overweight.

### Increased airway hyperreactivity after pHA despite unaltered bronchial structure

Beside the aforementioned functions, insulin and leptin promote fibrotic processes and increase contractility, contributing thereby to increased airway resistance^20^,^31^,^38^,^39^. In our study we report airway hyperreactivity despite unchanged bronchial or peripheral lung structure, suggesting that rather dysregulated bronchial smooth muscle cell homeostasis and thereby increased contractility underline the pro-asthmatic phenotype after pHA than altered bronchial or peripheral lung structure. Lung matrix remodeling in mice with asthma is partially characterized by increased collagen deposition and subepithelial fibrosis. We found that early-onset obesity leads to greater collagenIα1 abundance in the lung, which could favour profibrotic processes. Histological assessment of connective tissue in the lung, however, did not demonstrate fibrosis at P70. Probably this time point is too early to visualize fibrotic changes after pHA. Furthermore, lung matrix remodeling is not only related to increased deposition of collagen, but also to increased matrix stiffness and loss of elasticity. In the present study, elastin synthesis in the lung was dynamically regulated over time. Even though gene expression of tropoelastin was not regulated at P21 or P70, protein abundance was markedly increased at P21 and decreased at P70. Those results indicate that regulation of tropoelastin protein might not be regulated transcriptionally, but rather by posttranscriptional modifications. Various studies indicate that elastic fibers are predominantly synthesized during development and reduced in the ageing lung^34^,^35^,^55^. Quantification of elastic fibers in our study confirmed that there is a reduction of elastic fibers comparing P21 to P70 (supplemental online data). The lungs of the pHA^mouse^-group, however, tended to less decrease of elastic fibers over time. Since proteolytic activity was not different after pHA (supplemental online data) and mRNA expression of tropoelastin was not altered either when compared to Ctrl, we speculate that an increased deposition of tropoelastin at P21 could result in transient greater assembly of elastic fiber and ultimately less reduction over time when compared to Control (Ctrl). Another mechanism contributing to the temporo-dynamic regulation of tropoelastin and assembly of elastic fibers could be either a reduced crosslinking at P21 and increase P70 or a compensation of reduced tropoelastin abundance by other components of the elastic fiber, such as fibrillin1. But these ideas are speculative and require further investigation.

Overall this study demonstrates that early-onset obesity as a result of maternal HFD during lactation does not only dysregulate intrinsic-pulmonary STAT3-AMPKα-SOCS3, but also activate AKT/GSK3β pathway, indicative for insulin signaling. Those findings are coupled with increased expression of IL-1β and IL-23 in WAT, which in turn could result in increased expression of pulmonary cytokines, in particular IL-17A as a marker or TH-17 cells, at an early phase (P21). These early changes during lung development result in alterations of matrix composition favouring fibrosis and increased airway hyperreactivtiy in adulthood. In conclusion, this study gives initial evidence that “metabolic programming” through disruption of intrinsic pulmonary STAT3/AMPKα/SOCS3- and insulin signaling during a critical window of lung development contributes to a pro-asthmatic phenotype in adulthood and offers novel avenues to define preventive strategies.

## Materials and Methods

### Animal experiments

All animal procedures were performed in accordance with the German regulations and legal requirements and were approved by the local government authorities (Landesamt für Natur, Umwelt und Verbraucherschutz, NRW, Germany; AZ #84–02.04.2012A424 and AZ #8.87–50.10.37.09.292). Mice (C57B6N) from our own colony were housed in a room maintained at 22 ± 2 °C, exposed to a 12 hour dark/light cycle. *Primipara* female mice were fed regular standard show and mated at 10 weeks of age. After detection of vaginal plugging, dams continued on standard laboratory chow (SD) *ad libitum* (standard diet; ssniff #R/M-H, V1534-0). At day of birth dams were randomly assigned either to SD (Ctrl, Ctrl) or a high fat diet (HFD, pHA^mouse^ group) (modified #C1057, Altromin, Lage, Germany) for the lactation period [postnatal day 1 (P1) to P21]; litters size was normalized to six for each litters. Water was available *ad libitum* and food was withdrawn only for experimental reasons. After weaning at P21 male pups of both groups were fed SD until P70. At P21 and P70 Ctrl and pHA^mouse^ animals were sacrificed defining two groups: Ctrl and pHA^mouse^. Exact number of animals and original litterss are listed in the figure legends. Only male mice were included in the experiments to exclude gender differences.

### Physiological data of animals after early postnatal hyperalimentation (pHA)

Murine body weight in gram (g) was obtained at 5 different time points: P1, P7, P14, P21, and P70 by weighing each animal. The animals were colour marked for subsequent measurement over time.

### Leptin, insulin and IL-6 ELISA

Measurement of murine serum leptin, insulin and IL-6 levels was performed using commercial available ELISA systems according to the manufacturer’s instructions (mouse leptin ELISA, ESML-82K, Milipore; mouse insulin ELISA, EZRMI.13K, Milipore; Mouse IL-6 ELISA, M6000B, R&D Systems).

### Intraperitoneal glucose tolerance test

After weaning at P21 and at P70 male mice underwent an intraperitoneal (i.p.) glucose tolerance test (i.p. GTT) as previously described^56^. In brief, animals were fasted overnight (16h). Fasting blood glucose levels were determined, followed by i.p. injection of 20% glucose (10 ml/kg body weight). Blood glucose levels were measured using a glucometer (GlucoMen^®^ LX, A.Menarini diagnostics) after 15, 30, 60 and 120 minutes.

### Tissue preparation

The animals were sacrificed at P21 and at P70. Serum samples were frozen at −80 °C. Epigonadal fat pads containing WAT - indicator of whole body fat - were dissected and weighed; the right lung was removed and both were snap-frozen in liquid nitrogen. The left lung was inflated via tracheotomy and pressure-fixed at 20 cm H_2_O with 4% (mass/vol) paraformaldehyde, followed by paraffin embedding and sectioning as described previously^57^.

### Hematoxylin and eosin staining of lung sections

Murine lung tissue sections were stained with hematoxylin and eosin as described previously^57^.

### Staining and quantification of elastic fibers

To analyze elastic fiber density, as an index of parenchymal elastin content, quantitative lung morphology was performed. Ten random tissue sections of six animals per group, were analysed. Three-micrometer cross-sections of the lung were stained for elastin with Hart’s elastic stain (Weigert’s iron resorcin and fuchsin solution; Carl Roth, X877.3) and counterstained with tartrazine (dianova, #TZQ999). Elastic fiber density of alveoli and conducting airways was measured using Cell D 3.4 Olympus Soft Imaging Solutions (Olympus, Hamburg, Germany) and related to total lung tissue or bronchial wall surface, respectively.

### Picro sirius-red staining to visualize collagen deposition and fibrosis in the lung

To visualize connective tissue content picro sirius red staining was performed as described previously^58^. Three-micrometer cross-sections of the lung were stained using Picro Sirius Red Stain kit according to the manufacturer’s instructions (ScyTek Laboratories, Inc. U.S.A., SRC-1-IFU). Images were obtained using Cell D 3.4 Olympus Soft Imaging Solutions (Olympus, Hamburg, Germany).

### Histomorphometric analysis of bronchi and peripheral lung

For histomorphometric analysis of the murine lungs, the mean linear intercept (MLI) was measured by light microscopy using an Olympus BX 40 microscope (Olympus, Hamburg, Germany) on hematoxylin and eosin stained lung sections as described previously^57^. Briefly, six animals per group were analyzed, ten sections per animals were stained, five random fields were photographed with 20× magnifications, and 10 horizontal lines were drawn across each field. Large airways, vessels and atelectatic areas were avoided. Each intercept of the lines and alveolar walls was counted and the total number of intercepts per field was divided through the total length of lines. Pictures were acquired and evaluated using Cell D 3.4 Olympus Soft Imaging Solutions (Olympus, Hamburg, Germany).

### Quantitative assessment of bronchial smooth muscle layer (bSML), and bSML related to the bronchial wall thickness

To measure thickness of the bronchial smooth muscle layer (bSML) in lungs at P70, three-micrometer cross-sections were stained with mouse monoclonal antibody raised against smooth muscle, actin (Santa Cruz Biotechnology, Inc. #sc53142) as described previously^11^. Cell Sens dimensions Software (Olympus life science, Hamburg, Germany) was applied to perform quantitative assessment of bSML thickness. In brief, six animals per group and 20 bronchi per animal were measured, only transverse sliced bronchi were analysed. Thickness of the bSML was measured at three different positions and mean value of the three measurements was calculated as bSML thickness. The outer and the inner bronchial diameter were measured at the widest point and the bronchial wall thickness was calculated as the difference between both. Next, we assessed the ratio between thickness of the bSML and the bronchial wall thickness.

### RNA extraction, real time RT-PCR

Total RNA was isolated from frozen lung tissue and WAT at P21 and P70 as previously described^59^. Quantitative changes in mRNA expression were assessed by quantitative realtime-PCR as described previously using the 7500 Real-time PCR system (Applied Biosystem, Foster City, CA). The relative amount of the specific mRNA was normalized to Glycerinaldehyd-3-phosphat-Dehydrogenase (GAPDH) gene. Primer pairs and TaqMan probes used in this study are listed in the supplemental table 1.

### Protein isolation and immunoblotting

Protein extraction from total lung homogenate, gel electrophoresis and immunoblotting were performed as described previously^10^. Blots were probed with the following antibodies: monoclonal rabbit anti-phospho-AKT (Ser473, cell signaling, Danvers, MA, #4058, 1:1000), monoclonal rabbit anti-total AKT (cell signaling, Danvers, MA, #9272, 1:2000), monoclonal rabbit anti-phospho AMPKα (Thr172; cell signaling, Danvers, MA, #2535, 1:1000), monoclonal rabbit anti-total AMPKα (cell signaling, Danvers, MA, #2603, 1:3000), monoclonal rabbit anti-phospho GSK-3β (Ser9; cell signaling, Danvers, MA, #9336, 1:1000), monoclonal rabbit anti-total GSK-3β (cell signaling, Danvers, MA, #9315, 1:2000), monoclonal rabbit anti-INSULIN-RECEPTOR β (INS-R) (cell signaling, Danvers, MA, #3025, 1:1000), monoclonal rabbit anti-INSULIN-RECEPTOR SUBSTRATE 1 (IRS1) (cell signaling, Danvers, MA, #3407, 1:1000), monoclonal mouse anti-proliferating cell nuclear antigen (PCNA; DakoCytomation, Glostrup, Denmark #M 0879, 1:10 000), polyclonal rabbit anti-phospho-STAT3 (Tyr705, cell signaling, Danvers, MA, #9145, 1:1000), monoclonal mouse anti-total STAT3 (cell signaling, Danvers, MA, #9139, 1:2000), polyclonal rabbit anti-suppressor of cytokine signaling3 (SOCS3) (cell signaling, Danvers, MA, #2923, 1:1000), rabbit anti-COLLAGEN Iα1 (abcam, Boston, MA, #ab34710, 1:500), polyclonal goat anti-ELASTIN (Santa Cruz Biotechnology, Inc, Dallas, TX, sc-17581, 1:200); whereas monoclonal mouse anti-β-ACTIN (cell signaling, Danvers, MA, #3700, 1:3000), served as a loading Control (Ctrl). Anti-mouse IgG, HRP-linked (cell signaling, Danvers, MA, #7076, 1:2000), and anti-rabbit IgG, HRP-linked (cell signaling, Danvers, MA, #7074, 1:2000) were used as secondary antibodies.

For quantitative immunoblot analyses densitometry was performed using Bio-Rad ImageLab software (Bio-Rad, Munich, Germany), and values were normalized to β-ACTIN.

### Measurement of airway resistance (Res) and respiratory system compliance (Cdyn)

At P70 respiratory system resistance (Res), airway responsiveness and respiratory system compliance (Cdyn) were assessed with direct plethysmography for mice (FinePointe™RC; Buxco, Wellington, NC, USA). Mice were deeply anesthetized by intraperitoneal injection of ketamine (100 mg/kg body weight) and xylazine (5 mg/kg body weight), tracheotomized and ventilated. Airway resistance (Res) and Cdyn were measured at baseline after nebulization with PBS, followed by stimulation with methacholine–a bronchoconstrictor–in two different doses (6,25 mg/ml PBS and 12,5 mg/ml PBS) and measurement of Res to determine airway reactivity. In addition, we assessed minute volume (ml/min) and tidal volume (ml).

### Analysis of data

ΔΔCt– method was used to determine the expression level of each gene and expressed as fold induction. Values are shown as means ± standard error of the mean (SEM). Student t-test, Mann Whitney test, and Two way ANOVA followed by Bonferroni post-test were used to test significant differences. A p-value less than 0.05 was considered as significant. The procedures were carried out using the Graph Pad Prism software (GraphPad Software Version 4.0, San Diego, CA, USA).

## Additional Information

**How to cite this article**: Dinger, K. *et al.* Early-onset obesity dysregulates pulmonary adipocytokine/insulin signaling and induces asthma-like disease in mice. *Sci. Rep.* **6**, 24168; doi: 10.1038/srep24168 (2016).

## Supplementary Material

Supplementary Data
