# Supplementary material for: Early-onset obesity dysregulates pulmonary adipocytokine/insulin signaling and induces asthma-like disease in mice
Source: Sci Rep. 2016 Apr 18;6:24168. doi: 10.1038/srep24168 (PMC4834579; doi:10.1038/srep24168)
Supplement: Supplementary Data [file srep24168-s1.doc]

**Early-onset obesity dysregulates pulmonary adipocytokine/insulin signaling and induces asthma-like disease in mice**

^1^Katharina Dinger*, ^1^Philipp Kasper*, ^2^Eva Hucklenbruch-Rother, ^1,2^Christina Vohlen, ^3^Eva Jobst, ^2^Ruth Janoschek, ^2^Inga Bae-Gartz, ^4^Silke van Koningsbruggen-Rietschel, ^3^Christian Plank, ^5^Jörg Dötsch and ^1^Miguel Angel Alejandre Alcázar

*equally contributed

^1^Experimental Pulmonology, University Hospital for Pediatrics and Adolescent Medicine, Faculty of Medicine, University of Cologne, Cologne, Germany, ^2^Metabolism and Perinatal Programming, University Hospital for Pediatrics and Adolescent Medicine, Faculty of Medicine, University of Cologne, Cologne, Germany, ^3^Department of Pediatrics and Adolescent Medicine, University Hospital Erlangen, Erlangen, Germany, ^4^Pediatric Pulmonology, University Hospital for Pediatrics and Adolescent Medicine, Faculty of Medicine, University of Cologne, Cologne, Germany, ^5^University Hospital for Pediatrics and Adolescent Medicine, Faculty of Medicine, University of Cologne, Cologne, Germany.

**Corresponding author:**

Miguel Angel Alejandre Alcázar M.D., Ph.D.

Department of Pediatrics and Adolescent Medicine, University of Cologne

Kerpener Strasse 62

50937 Cologne, Germany

Tel.: +49 (0) 221 478 96876

Fax.: +49 (0) 221 478 96868

Email: miguel.alejandre-alcazar@uk-koeln.de

**Online Supporting Material**

**Material and Methods**

*Rat model of early postnatal hyperalimentation induced by litter size reduction*

All procedures performed on animals were approved by the local government authorities (Regierung von Mittelfranken, AZ # 621-2531.31-11/02 and AZ # 621-2531.31-14/05 and Regierung von Nordrhein-Westphalen AZ # 8.87-50.10.37.09.292). Virgin female Wistar rats were obtained from our own colony and were housed in a room maintained at 22 ± 2°C, exposed to a 12 hour dark/light cycle. The animals were allowed unlimited access to standard diet (SD; #1320, Altromin, Lage, Germany) and tap water. The litters were either reduced to 10 (Ctrl^rat10^) or 6 (Ctrl^rat6^) pups on the first day of life. Each group (Ctrl^rat10^ and Ctrl^rat6^) was formed of males from 3-4 different litters. During lactation dams continued on SD. The weaning was at postnatal day 23 (P23). We included home-cage-control (Ctrl^HCC^, mean litter size of 16) animals without any postnatal manipulation during lactation, dams were fed SD as well. Subsequent weaning all three groups continued on SD. HCC, LSR10 and LSR6 animals were sacrificed at P70 defining 3 groups: HCC (5 animals, 3 dams), LSR10 (10 animals, 4 dams), LSR6 (8 animals, 3 dams). Body weight of the offspring was measured at P21 and P70.

*Measurement of airway resistance (Res) in the rat model*

At the age of P70, airway responsiveness was assessed by measuring respiratory system resistance (Res) with direct plethysmography for rats (FinePointe™ RC; Buxco, Wellington, NC, USA). Rats were deeply anesthetized by intramuscular injection of ketamine (100 mg/kg body weight) and midazolame (5 mg/kg body weight), tracheotomised and ventilated. Res was measured at baseline, after exposure to phosphate buffered saline (PBS), and after stimulation with increasing concentrations of methacholine (6.25, 12.5, 25 mg/ml), a bronchoconstrictor.

*Measurement of respiratory system compliance (Cdyn) in the murine model*

At P70 respiratory system compliance (Cdyn) was assessed with direct plethysmography for mice (FinePointe™RC; Buxco, Wellington, NC, USA). Mice were deeply anesthetized by intraperitoneal injection of ketamine (100 mg/kg body weight) and xylazine (5 mg/kg body weight), tracheotomized and ventilated. Cdyn was measured at baseline after nebulization with PBS.

*Protein isolation and immunoblotting*

Protein extraction from total lung homogenate, gel electrophoresis and immunoblotting were performed as described in the manuscript. Blots were probed with polyclonal rabbit anti-suppressor of cytokine signaling 3 (SOCS3); monoclonal mouse anti-β-ACTIN (cell signaling, Danvers, MA, #3700), served as a loading control. Anti-rabbit IgG, HRP-linked (cell signaling, Danvers, MA, # 7074, 1:2000) was used as secondary antibody. For quantitative immunoblot analysis densitometry was performed using Bio-Rad ImageLab software (Bio-Rad, Munich, Germany), and values were normalized to β-ACTIN.

*Gelatin zymography*

Gelatin zymography to assess MMP-2 activity was performed. Briefly, lung homogenate was incubated with non-denaturating sample buffer (62.5 mM Tris-HCl pH 6.8, 10% glycerol, 2% SDS, 0.0025% bromphenol blue) for 10 minutes at 37°C and then loaded onto 10% SDS-polyacrylamide gels containing 0.1% gelatin, followed by gel electrophoresis, washing for 30 minutes in renaturation buffer (2.5% Tx-100) and for 30 minutes in developing buffer (50 mM Tris-HCl pH 8, 0.2 M NaCl, 5 mM CaCl2, 0.02% Brij 35). After developing of the gel over night at 37°C gels were stained with 0.1% Coomassie brilliant blue in 25% isopropanol and 10% acetic acid. Imaging was performed with the Biodoc analyze system (Biometra).

**Results**

*Early postnatal hyperalimentation (pHA) with transient early-onset overweight does not alter alveolar size or respiratory system compliance (Cdyn) at P70 in mice*

To analyze whether a dysregulation of insulin and adipocytokine signaling leads to long-term changes of lung growth, we assessed mean linear intercept (MLI) as a parameter of alveolar size, but did not find a difference between the two groups, pHA^mouse^ and Ctrl (Fig. 1A, 1B). We next investigated whether a dysregulation of insulin and adipocytokine signaling leads to long-term changes of respiratory system resistance (Cdyn). Assessment of respiratory dynamic compliance (Cdyn) to determine elasticity of the lung did show a slight, but not significant increase (supplemental figure 1C).

*Early pHA with early-onset obesity leads to mild temporo-dynamic regulation of elastic fiber content in the lung in the murine offspring*

Since elastin is an important component of elastic fibers, main regulator of elasticity of the lung, we performed Hart’s staining using tartrazine as counterstain. Representative images depicting elastic fibers (red arrows) in lungs of the pHA^mouse^ and Ctrl-group at P21 and P70 indicated that lungs after pHA appear to have more elastic fibers than Ctrl (Fig 2A). We next quantified elastic fiber content per lung tissue excluding major vessels. P21 served as reference point and amount of elastic fibers at P70 was related to P21 in both groups pHA^mouse^ and Ctrl individually. We found a decrease of elastic fibers in both groups, but the reduction of elastic fibers in the pHA^mouse^-group tended to be less (p=0.1) (Fig 2A). We also assessed proteolytic activity of metalloproteinase-2 (MMP-2) and of MMP-9 (data not shown) by gelatine zymography, but did not find differences between pHA and Ctrl at P21 or at P70 (Fig. 2B).

*Effect of early-onset overweight on airway resistance in the rat model of pHA*

To test whether the effects of pHA on airway responsiveness is independent of species and maternal diet, litter size reduction during lactation was performed in a rat model and compared to the murine results. To induce pHA litter size was reduced directly after birth to 6 pups (pHA^rat6^) in comparison to the control group of litter size of 10 pups (Ctrl^rat10^). pHA^rat6^ group expressed a significantly greater body weight at P21 than the Ctrl^rat10^ group [supplemental figure 3A; (p=0.05)]. At P70, no body weight difference between pHA^rat6^group and Ctrl^rat10^group was detectable (supplemental figure 3B). The body weight of these animals was previously published in an article focussing on renal outcome (Alejandre Alcazar MA et al., Endocrinology, 2012). We next measured airway resistance and found that pHA^rat6^group exhibited a significant airway hyperreactivity after methacholine stimulation compared to Ctrl^rat10^group (supplemental figure 4). Taken together, early postnatal overweight as a result of pHA by litter size reduction in rats leads to a similar effect in the offspring as high fat diet feeding during lactation in the murine model.

*Effect of early postnatal overweight in the rat on pulmonary SOCS3 protein abundance*

Assessment of SOCS3 in total lung homogenate by immunblot showed a significant increase in the group with litter size reduction to 6 pups (pHA^rat6^) when compared to the HCC control group (p<0.01) (supplemental figure 5), indicative of activated leptin/IL-6 signaling in the lung.

**
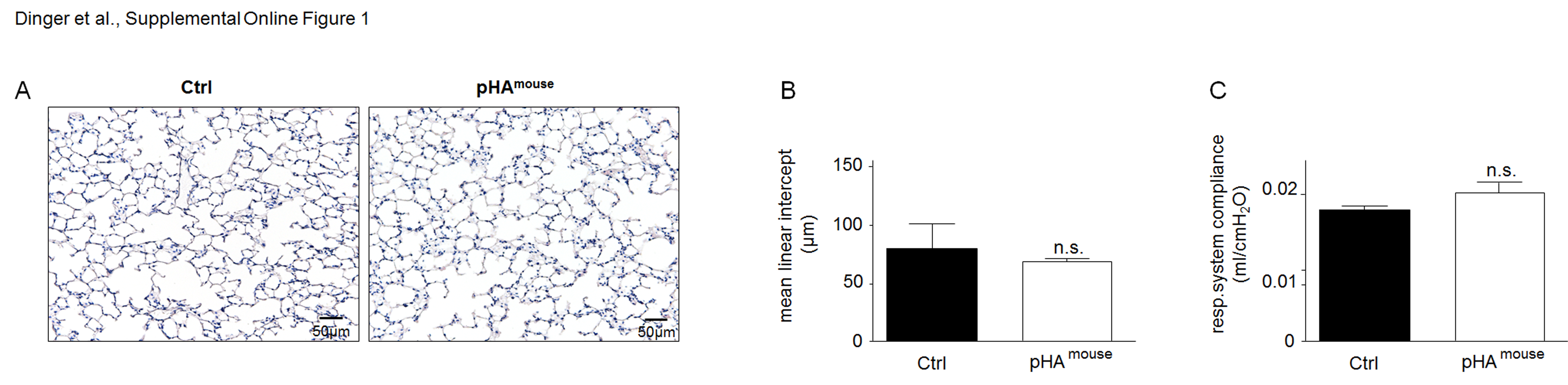
**

**Supplemental Figure 1.** *Early postnatal hyperalimentation (pHA) does not alter alveolar size or respiratory system compliance (Cdyn) in the murine model at postnatal day (P70).*

(A) Representative images of hematoxylin and eosin (H&E) stained lungs sections at postnatal day (P70). (B) Assessment of alveolar size at P70 using quantitative image analysis of mean linear intercept (MLI). Ctrl: n = 5 from 2 litter; pHA^mouse^: n = 5 from 2 litter (C) Measurement of respiratory system compliance (Cdyn). Early postnatal hyperalimentation (pHA^mouse^group, n = 14 from 4 litter; open square) compared to the control group (Ctrl, n = 9 from 6 litter; solid square). Mean ± SEM; Mann-Whitney test; n.s. = not significant.

**
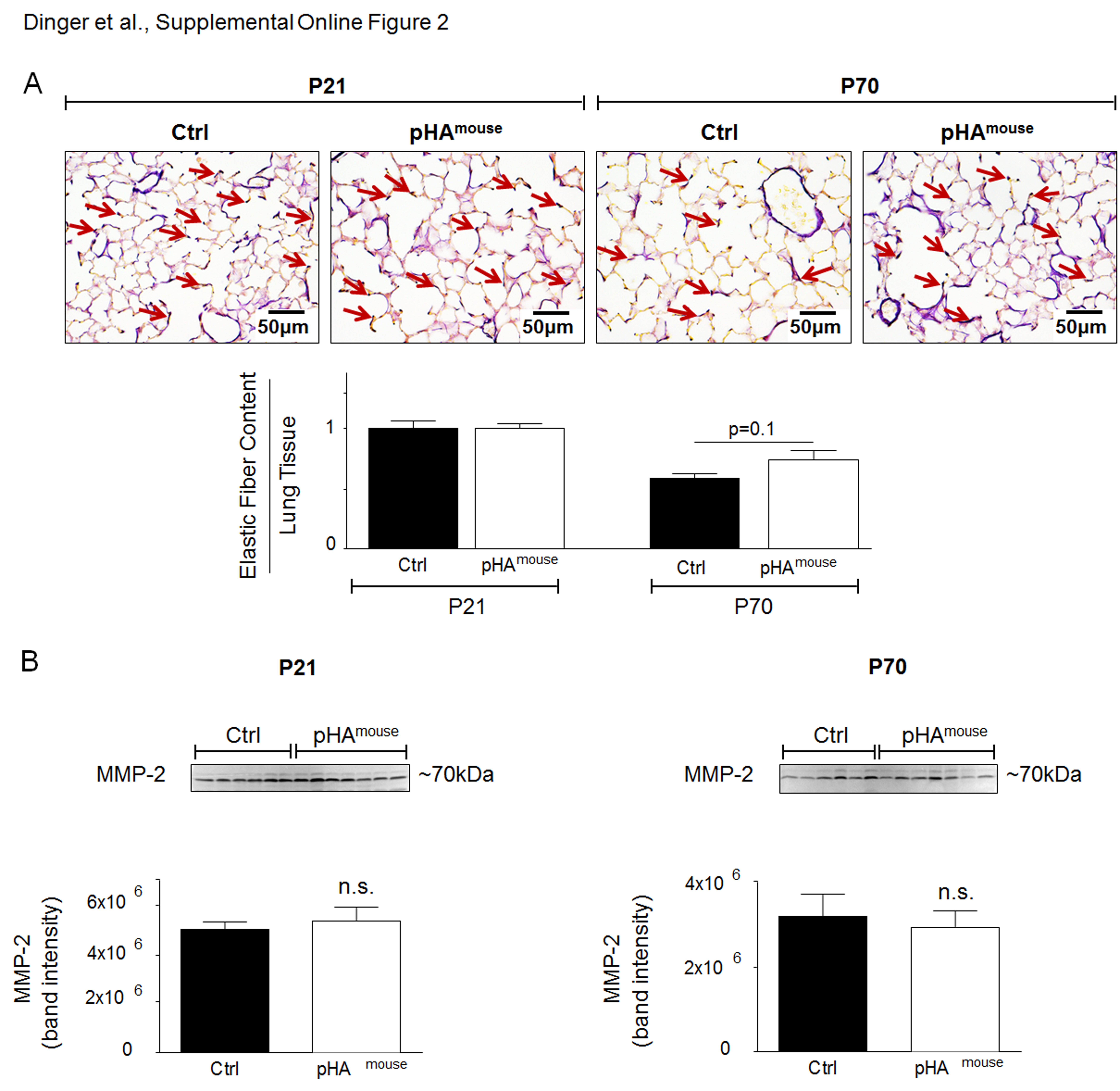
**

**Supplemental Figure 2**. *Early postnatal hyperalimentation (pHA) does neither significantly regulate elastic fiber content in the peripheral lung nor affect lung metalloproteinase 2 (MMP-2) activity.*

(A) Representative images illustrating elastic fibers using Hart’s staining in paraffin-embedded and paraformaldehyde-fixed lungs at P21 (left panel) and P70 (right panel). Red arrows are depicting positive staining of elastic fibers. Quantification of elastic fibers per lung tissue is below the images. Elastic fiber content at P21 was set as 1 and elastic fiber content at P70 was related to P21 Ctrl: n = 6 from 5 litter; pHA^mouse^: n = 6 from 4 litter. (B) Assessment of proteolytic activity of metalloproteinase-2 (MMP-2) in total lung homogenate at P21 (left panel) and P70 (right panel) using gelatine zymography. Images are inverted and digested gelatin is shown in black. Densitometric analysis of absolute band intensity is shown below the respective zymograph. Ctrl: n = 6 from 4 litter; pHA^mouse^: n = 7 from 4 litter. Early postnatal hyperalimentation (pHA^mouse^group) compared to the control group (Ctrl). pHA^mouse^group: white bar; Ctrl: black bar. Mean ± SEM; Mann-Whitney test; n.s. = not significant.

**
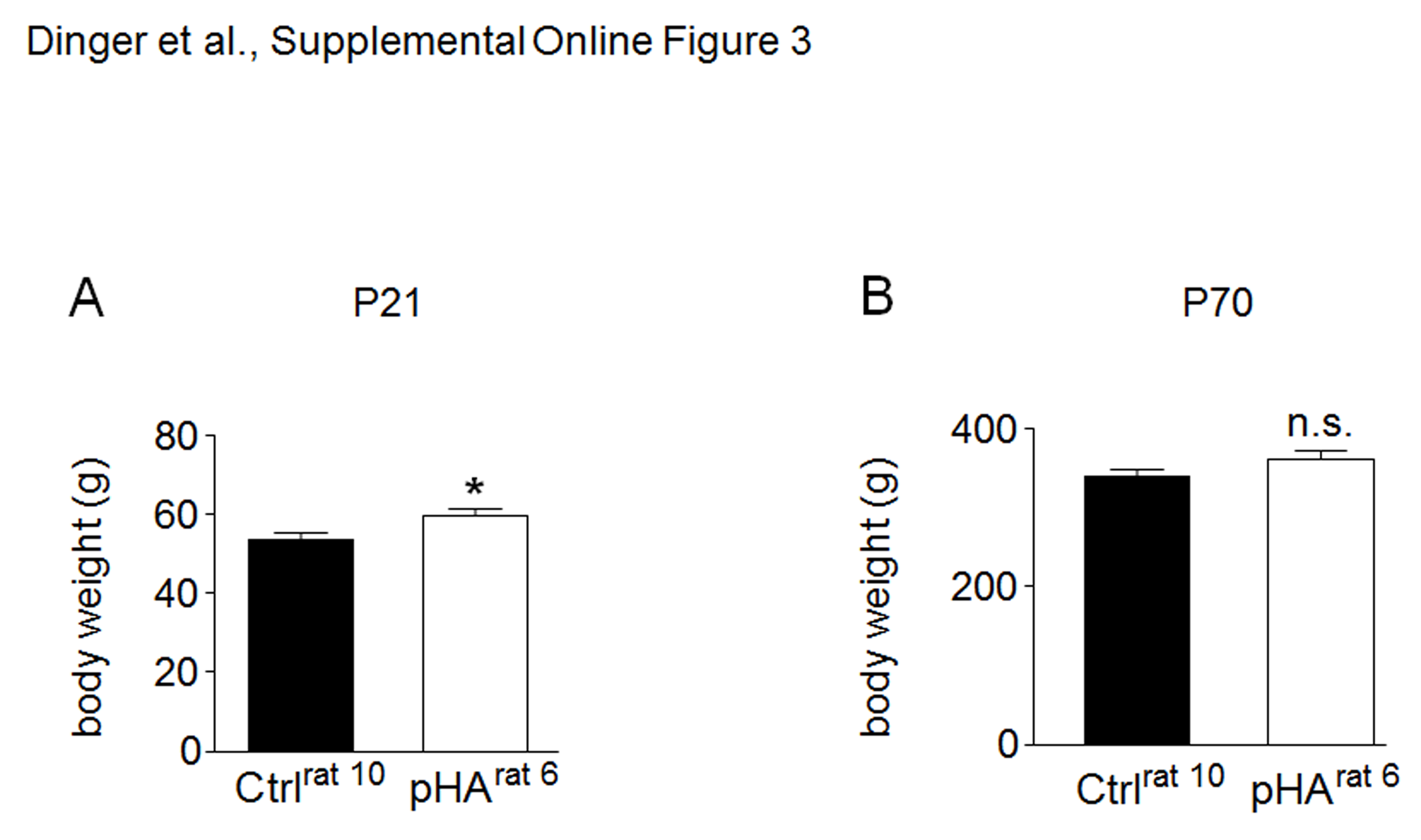
**

**Supplemental Figure 3.** *Litter size reduction induces early postnatal hyperalimentation (pHA) with early-onset overweight in a rat offspring.*

A, B: Body weight (gram) at P21 (A) and P70 (B) in a rat model of litter size reduction to induce early postnatal hyperlimentation (pHA) with early-onset overweight. Litter size reduction to 6 pups (pHA^rat6^ group; white bar), control group with litter size of 10 (Ctrl^rat10^group; black bar), n = 8-10/group.

**
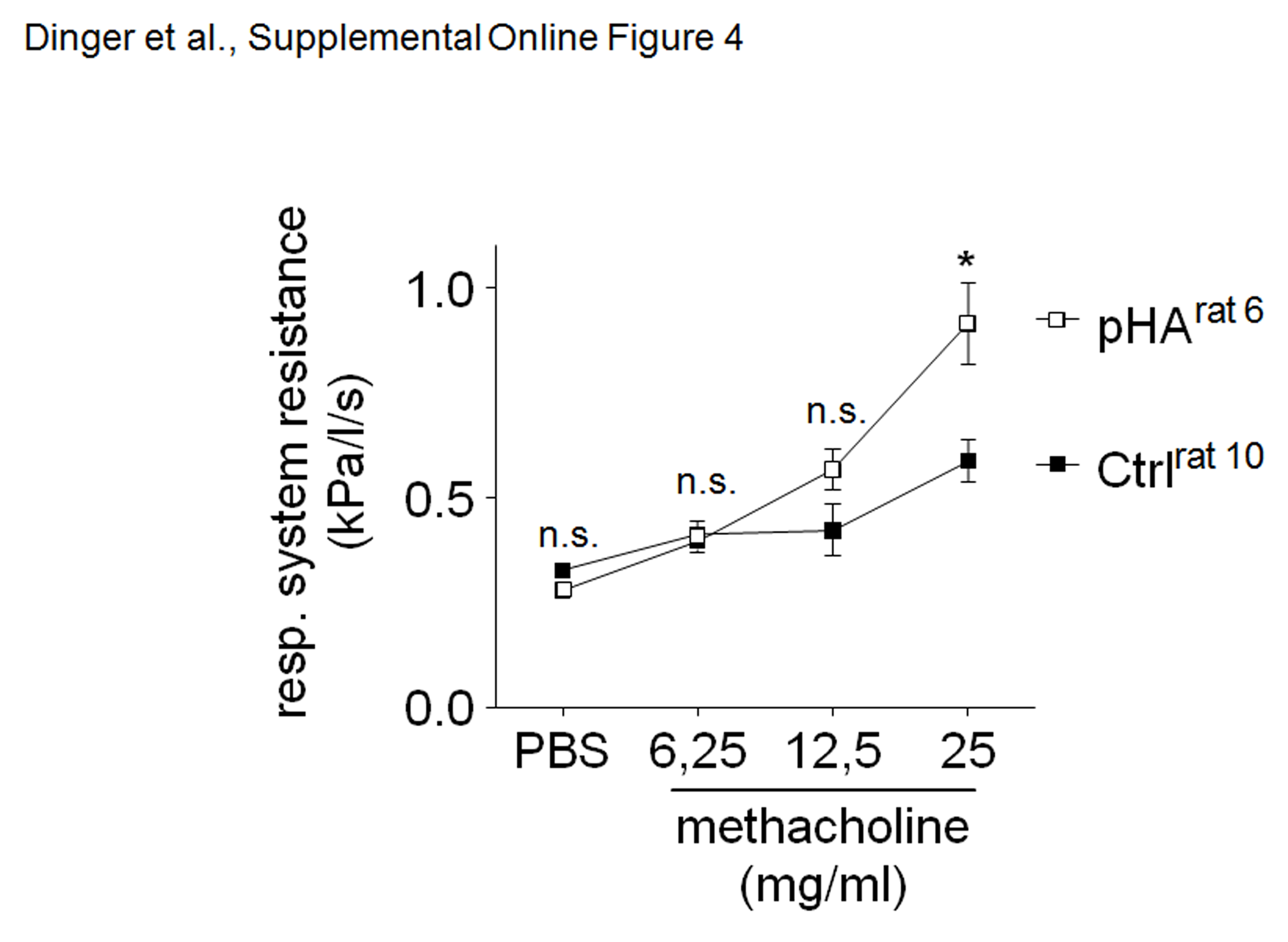
**

**Supplemental Figure 4.** *Early postnatal hyperalimentation (pHA) increases airway resistance in the rat model at postnatal day 70 (P70).*

Airway resistance (Res) after methacholine stimulation measured by direct plethysmography. Exposure of the rats to PBS, followed by increasing doses of methacholine: 6.25mg/ml and 12.5mg/ml, 25mg/ml . Res was significantly increased after methacholine stimulation at P70 in the group with early postnatal hyperalimentation (pHA) induced by litter size reduction to 6 (pHA^rat6^group) compared to the control group with litter size reduction to 10 (Ctrl^rat10^group). pHA^rat6^group: white square; Ctrl^rat10^group: black square. Mean ± SEM; n = 3-10; two-way ANOVA and Bonferroni posttest. *p<0.05, n.s. = not significant.

**
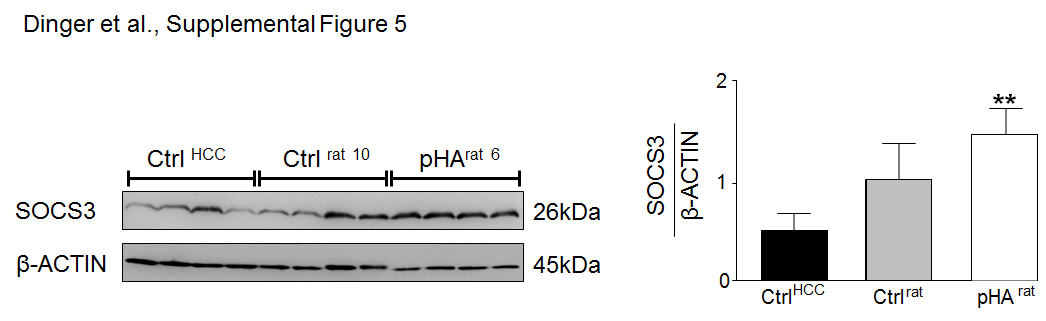
**

**Supplemental Figure 5.** *Early postnatal hyperalimentation (pHA) increases suppressor of cytokine signaling 3 (SOCS3) protein abundance in lungs of rats at postnatal day 70 (P70).*

Immunoblot showing suppressor of cytokine signaling 3 (SOCS3) protein in lungs of rats after postnatal hyperalimentation (pHA) induced by litter size reduction to 10 (Ctrl^rat10^group) or 6 (pHA^rat6^group) at birth compared to the control group without any litter size reduction (home-caged control, Ctrl^HCC^, average litter size 12-14 pups per dam); Ctrl^HCC^: black bar; Ctrl^rat10^group: grey bar; pHA^rat6^group: white bar. Mean ± SEM; n = 4/group; one-way ANOVA and Bonferroni posttest; **p<0.01.

**Supplemental Table 1**

| ***Gapdh*** | forward 5’-ACGGGAAACCCATCACCAT-3’  reverse 5’-CCAGCATCACCCCATTTGA-3’  probe 5‘(FAM)-TTCCAGGAGCGAGATCCCGTCAAG-(TAMRA)3‘ |
| --- | --- |
| ***Il-6*** | forward 5’-TCCAAACTGGATATAACCAGGAAAT-3’  reverse 5’-TTGTCTTTCTTGTTATCTTGTAAGTTGTTCTT-3’  probe 5‘(FAM)-AATCTGCTCTGGTCTTCTGGAGTTCGGTTTCTA-(TAMRA)3‘ |
| ***tropoelastin*** | forward 5’- CTACGGACTGCCCTATACCAATG-3’  reverse 5’-CACCATACTTGGCTGCTTTAGCT-3’  probe 5‘(FAM)-CAAGGCTGGCTACCCAACAGGGACA-(TAMRA)3‘ |

| ****Gapdh*** | forward 5’-TGTGAAGCTCATTTCCTGGTATGA-3’  reverse 5’-CTCTCTTGCTCTCAGTATCCTTGCT-3’ |
| --- | --- |
| ****Il-1β*** | forward 5’-TGACAGTGATGAGAATGACCTGTTC-3’  reverse 5’-GGACAGCCCAGGTCAAAGG-3’ |
| ****Il-4*** | forward 5’-GGAGATGGATGTGCCAAACG-3’  reverse 5’-GCACCTTGGAAGCCCTACAG-3’ |
| ****Il-13*** | forward 5’-GCTTATTGAGGAGCTGAGCAACA -3’  reverse 5’-CCAGGTCCACACTCCATACCA-3’ |
| ****Il-17A*** | forward 5’-CCAGAAGGCCCTCAGACTACCT-3’  reverse 5’-GGGATATCTATCAGGGTCTTCATTG-3’ |
| ****Il-23*** | forward 5’-TGACCCACAAGGACTCAAGGA-3’  reverse 5’-CCCTTTGAAGATGTCAGAGTCAAG-3’ |
| ****Tnf-α*** | forward 5’-AGGGATGAGAAGTTCCCAAATG-3’  reverse 5’-GCTTGTCACTCGAATTTTGAGAAG-3’ |

**Supplemental Table 1**: Designed TaqMan primers and Sybr green primers (*). Interleukin-1β, *Il-1β*; Interleukin-4, *Il-4*; Interleukin-6, *II-6*; Interleukin-13, *Il-13*; Interleukin-17A, *Il-17A*; Interleukin-23, *Il-23*; Tumor necrosis factor-alpha (*Tnf-α*); *tropoelastin*.
